# Reduced expression of proteolipid protein 2 increases ER stress‐induced apoptosis and autophagy in glioblastoma

**DOI:** 10.1111/jcmm.14840

**Published:** 2019-11-28

**Authors:** Zichao Feng, Wenjing Zhou, Jiwei Wang, Qichao Qi, Mingzhi Han, Yang Kong, Yaotian Hu, Yulin Zhang, Anbin Chen, Bin Huang, Anjing Chen, Di Zhang, Wenjie Li, Qing Zhang, Rolf Bjerkvig, Jian Wang, Frits Thorsen, Xingang Li

**Affiliations:** ^1^ Department of Neurosurgery Qilu Hospital of Shandong University Shandong Key Laboratory of Brain Functional Remodeling and Brain Science Research Institute Shandong University Shandong China; ^2^ Department of Biomedicine University of Bergen Bergen Norway; ^3^ Department of Oncology Luxembourg Institute of Health Luxembourg Luxembourg; ^4^ The Molecular Imaging Center Department of Biomedicine University of Bergen Bergen Norway

**Keywords:** apoptosis, autophagy, ER stress, glioblastoma, proteolipid protein 2

## Abstract

Proteolipid protein 2 (PLP2) is an integral ion channel membrane protein of the endoplasmic reticulum. The protein has been shown to be highly expressed in many cancer types, but its importance in glioma progression is poorly understood. Using publicly available datasets (Rembrandt, TCGA and CGGA), we found that the expression of *PLP2* was significantly higher in high‐grade gliomas than in low‐grade gliomas. We confirmed these results at the protein level through IHC staining of high‐grade (n = 56) and low‐grade glioma biopsies (n = 16). Kaplan‐Meier analysis demonstrated that increased *PLP2* expression was associated with poorer patient survival. In functional experiments, siRNA and shRNA PLP2 knockdown induced ER stress and increased apoptosis and autophagy in U87 and U251 glioma cell lines. Inhibition of autophagy with chloroquine augmented apoptotic cell death in U87‐ and U251‐siPLP2 cells. Finally, intracranial xenografts derived from U87‐ and U251‐shPLP2 cells revealed that loss of PLP2 reduced glioma growth in vivo. Our results therefore indicate that increased PLP2 expression promotes GBM growth and that PLP2 represents a potential future therapeutic target.

## 1 INTRODUCTION

Gliomas constitute approximately 75% of the malignant primary brain tumours in adults.^1^ The life expectancy of patients with glioblastoma (GBM, WHO grade IV) is on average 14 months after diagnosis, and current treatment strategies have shown only limited survival benefits.^2^ Consequently, a deeper understanding of the mechanisms underlying glioma growth and progression is important in order to develop new therapeutic strategies.

The proteolipid protein 2 (*PLP2*) gene, also known as *A4/A4LSB,* was first discovered in colon epithelial cells. While the exact function of PLP2 under normal conditions is not known, the study of the protein has revealed several features. First, it is an integral membrane protein that localizes to the endoplasmic reticulum (ER). Second, it has been shown to multimerize and to exhibit ion channel characteristics.^3^ Third, *PLP2*‐knockout mice display increased ER stress in neurons under hypoxia, which leads to apoptotic cell death.^7^ Finally, PLP2 might have a role in normal gastrulation.^8^ PLP2 has also been reported to be involved in disease. In several cancers, for example, such as melanoma, breast cancer and osteogenic sarcoma, the protein has been shown to promote cell growth, proliferation and migration.^4^, ^5^, ^6^

Around one‐third of the total proteome is synthesized in the ER which is the dominating subcellular compartment involved in protein folding and maturation.^9^, ^10^ Various physiological and pathological stimuli can alter ER function, which leads to ER stress. In many instances, ER stress leads to an accumulation of unfolded or misfolded proteins inside the ER. For instance, ER stress can cause an unfolded protein response (UPR). Unfolded protein response is an adaptive reaction to reduce the unfolded protein load in order to maintain cellular viability and function.^11^ Therefore, under ER stress, the accumulation of misfolded proteins triggers UPR, which leads to an activation of biochemical mechanisms alleviating ER stress. If homeostasis cannot be restored by UPR, autophagy or apoptotic cell death may be activated.^12^, ^13^

Autophagy is involved in the delivery of unwanted cytoplasmic cargo to the lysosomes for subsequent degradation within autophagosomes. It is essential for survival and homeostasis and may mediate resistance to anticancer therapies such as radiation, chemotherapy and targeted therapies.^14^, ^15^ However, the role of autophagy in cancer is still inconclusive, since under some conditions, autophagy suppresses tumorigenesis,^16^ whereas in others, it facilitates tumour growth.^17^

Apoptosis is a highly regulated form of programmed cell death. A better understanding of the signalling pathways that control apoptosis in different tumour types has been important for the discovery of novel targeted agents and for the design of clinical trials.^18^, ^19^ As a result, apoptosis and the induction of autophagy, and in particular the crosstalk between these two processes, remain a subject of interest.

In the present study, we show in both clinical samples and cell lines that PLP2 is significantly overexpressed in GBMs compared with normal human astrocytes (NHAs) and normal human brain tissue samples. We investigated the significance of this finding through PLP2 knockdown in U87 and U251 glioma cell lines in vitro and in vivo. We found that PLP2 knockdown reduces cell proliferation and increases ER stress‐induced apoptosis and autophagy via CCAAT‐enhancer‐binding protein homologous protein (CHOP), a ubiquitous transcription factor known to have a regulatory role in the crosstalk between autophagy and apoptosis.^20^ Our results therefore establish a vital role for PLP2 in promoting GBM growth and as a potential therapeutic target.

## 2 RESULTS

### 2.1 PLP2 overexpression is related to glioma tumour grade and patient prognosis

**Figure 1:**
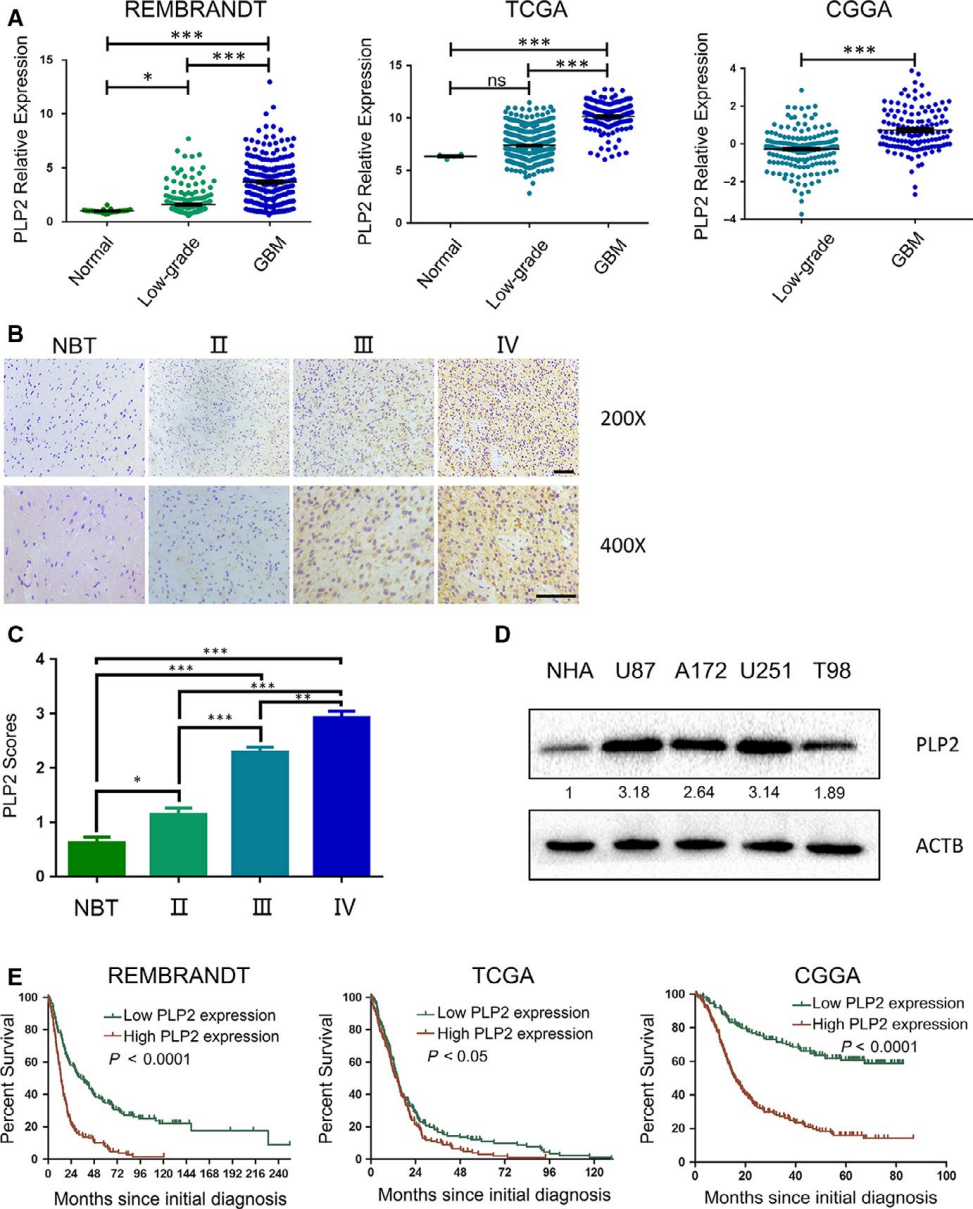
PLP2 expression is up‐regulated in high‐grade gliomas and inversely associated with glioblastoma patient prognosis. A, Relative expression levels of *PLP2* mRNA in samples analysed in publicly available databases REMBRANDT, TCGA and CGGA. Ns, not significant, *P < .05 and ***P < .001. B, Representative images of immunohistochemical staining for PLP2 in 4‐µm sections from normal brain tissue samples (NBT), grade II gliomas (II), grade III gliomas (III) and glioblastomas (IV). Scale bar = 100 μm. C, Immunohistochemical scores for PLP2 in normal brain tissue samples (NBT), grade II gliomas (II), grade III gliomas (III) and glioblastomas (IV). Five random fields from each section were counted. Data are shown as the mean ± SD. *P < .05, **P < .01 and ***P < .001. D, Western blot analysis of PLP2 levels in NHA, U87, A172, U251 and T98 cell lines. E, Kaplan‐Meier analyses showing differences in overall survival for patients with low and high *PLP2* expressing gliomas. The data were obtained from Rembrandt, TCGA and CGGA databases

We first examined the levels of *PLP2* expression in human glioma samples by analysing publicly available datasets from Rembrandt, The Cancer Genome Atlas (TCGA) and the Chinese Glioma Genome Atlas (CGGA). *PLP2* was significantly up‐regulated in GBMs compared with low‐grade gliomas (Figure 1A, *P* < .001), as shown previously.^21^ To further determine whether PLP2 expression was associated with aggressive clinicopathological characteristics, PLP2 immunohistochemical (IHC) staining was performed on 72 paraffin‐embedded glioma tissue samples, including grade II (n = 16), III (n = 21) and IV (n = 35) brain tumour samples and normal brain tissue (n = 5) samples (Figure 1B). Quantitative analyses of mean PLP2 IHC staining scores showed that the PLP2 levels increased with disease grade (Figure 1C). Next, we performed Western blot analysis to determine PLP2 protein levels in normal human astrocytes (NHA) and human GBM cell lines U87, A172, U251 and T98. All cell lines displayed higher PLP2 protein levels compared with NHAs (Figure 1D). Furthermore, based on Kaplan‐Meier analysis of publicly available datasets (Rembrandt, TCGA and CCGA), high *PLP2* expression predicted a shorter overall patient survival (Figure 1E). Taken together, these results indicate that PLP2 has an important role in glioma progression.

### 2.2 Down‐regulation of PLP2 inhibits cell proliferation in glioma cells

**Figure 2:**
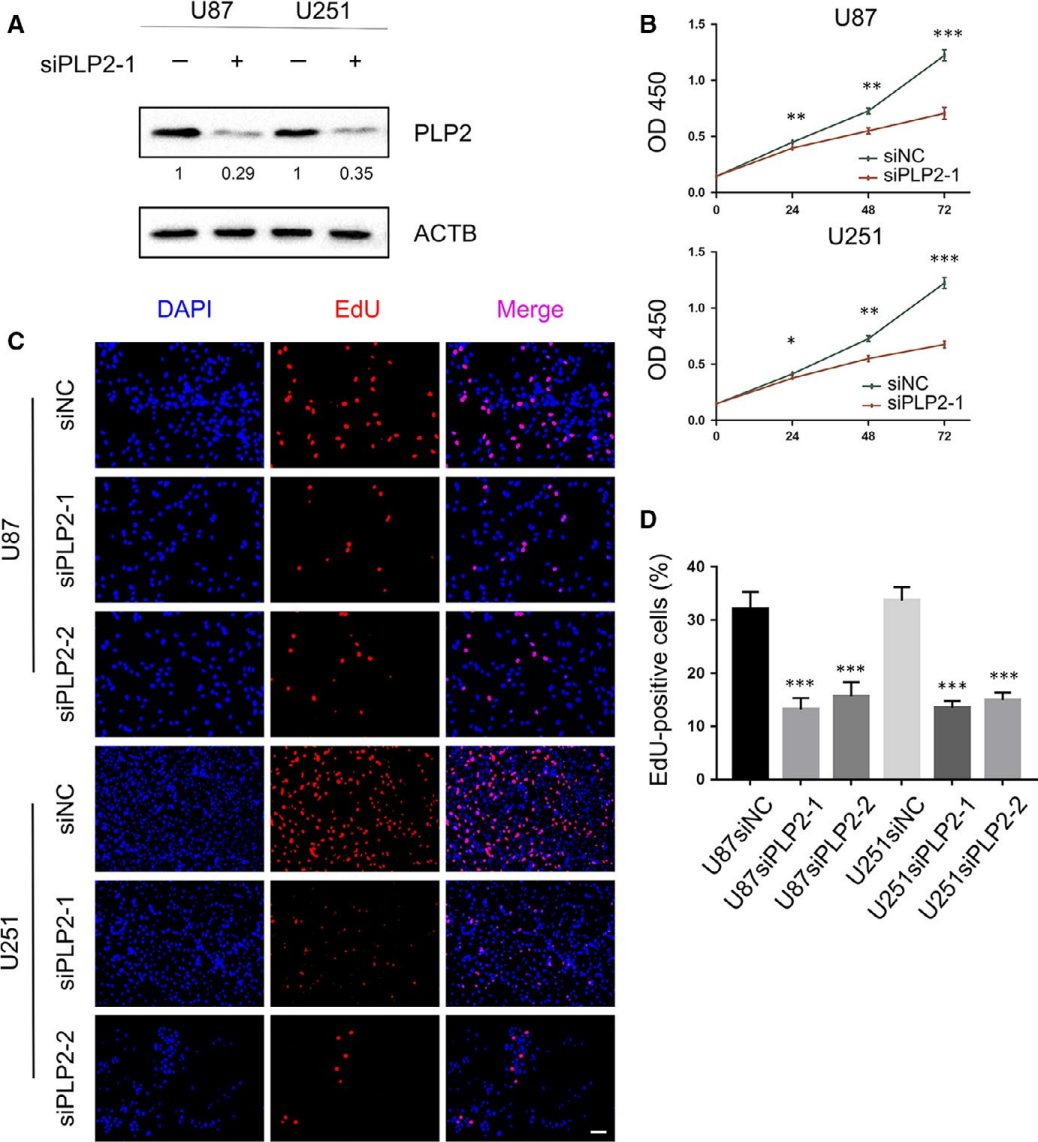
Down‐regulation of PLP2 expression inhibits glioma cell proliferation. A, Western blot to confirm knockdown efficiency of PLP2 by siRNA in U87 and U251 cells. B, Cell viability as determined using the CCK‐8 of U87 and U251 transfected with siPLP2 and the scrambled negative control, siNC. *P < .05, **P < .01 and ***P < .001. C, Fluorescence microscopy of EdU incorporation in U87 and U251‐siPLP‐1,2 siRNAs. Pollo 567 (red colour) detects EdU, while DAPI (blue colour) stains nuclei Scale bar = 50 μm. D, Statistical analysis of the number of EdU‐positive cells for U87 and U251 cell lines transfected with siRNAs. All data are expressed as the mean ± SD of values from triplicate experiments. ***P < .001

Several reports have shown that PLP2 is highly expressed in extracranial cancers where it has been shown to promote tumour growth and metastasis.^4^, ^22^, ^23^ To assess the biological role of PLP2 in glioma, we knocked down PLP2 expression in U87 and U251 GBM cells, using two small interfering RNAs (siPLP2‐1 and siPLP2‐2). Significant knockdown of PLP2 expression levels was obtained (Figure 2A and Figure S2A). Knockdown with siPLP2 also inhibited cell proliferation in both cell lines, as assessed in growth curves generated using the CCK‐8 assay (Figure 2B and Figure S2B). These results were verified using EdU incorporation, which revealed reduced proliferation in U87 and U251 transfected with siPLP2 compared with controls (Figure 2C,D).

### 2.3 Down‐regulation of PLP2 induces ER stress and promotes apoptosis mediated in part by CHOP

**Figure 3:**
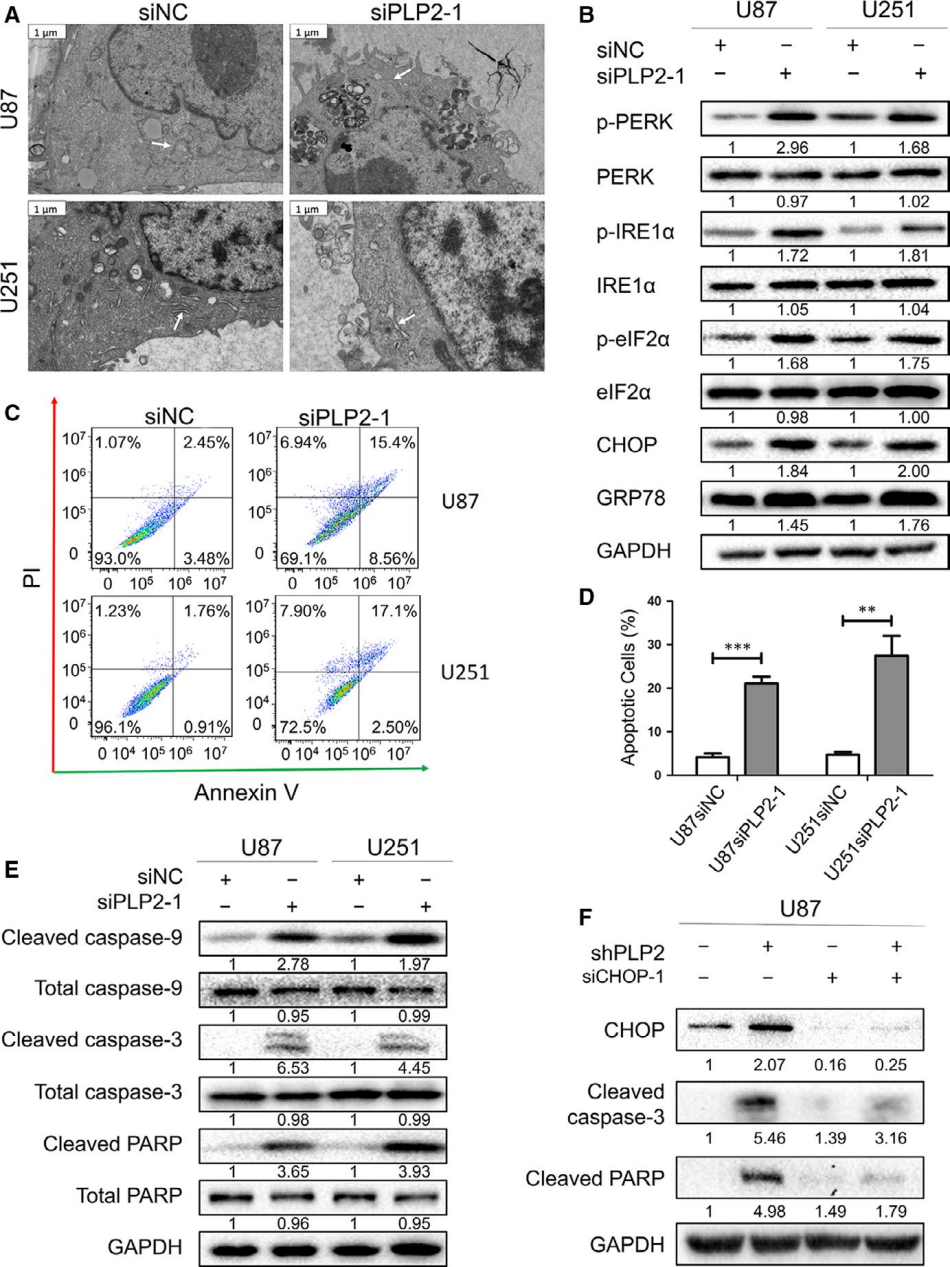
Down‐regulation of PLP2 expression induces ER stress and promotes apoptosis through CHOP in glioma cells. A, Transmission electron microscopy images showing dilated ER in U87 and U251 cells transfected with siPLP2‐1 for 48 h (right) compared with control siNC‐transfected cells. Examples of ER are highlighted by white arrows. Scale bars = 1 μm. B, Western blots showing the levels of ER stress‐related markers p‐PERK, PERK, p‐IRE1α, IRE1α, p‐eIF2α, eIF2α, CHOP and GRP78 in U87‐ and U251‐siNC or siPLP2‐1 cells. C, Flow cytometry detecting apoptosis in U87‐ and U251‐siNC or U251‐siPLP2‐1 at 48 h after transfection. D, Quantification of the percentage of apoptotic cells analysed by flow cytometry. **P < .01 and ***P < .001. E, Representative Western blots showing the levels of apoptosis‐related markers caspase‐9, caspase 3 and PARP in U87‐ and U251‐siNC or ‐siPLP2‐1 at 48 h after transfection. GAPDH is used as the control for loading. F, Western blots showing the levels of cleaved‐caspase3 and cleaved‐PARP levels in U87‐shNC and U87‐shPLP2 cells transfected with siNC or siCHOP‐1. GAPDH is used as the control for loading

Reduced PLP2 expression has previously been shown to increase ER stress‐induced neuronal apoptosis.^7^ To study potential changes in ER structures, we performed transmission electron microscopy (TEM) on U87 and U251 cells transfected with siPLP2 for 48 hours. The lumen of the ER was markedly dilated and fragmented in siPLP2‐treated cells compared with the control siNC transfected cells (Figure 3A and Figure S3A). To examine these changes at the molecular level, we performed Western blotting for ER stress‐related proteins in siPLP2 transfected cells relative to controls. Levels of ER stress‐related proteins, including phosphorylated protein kinase‐like endoplasmic reticulum kinase (p‐PERK), phosphorylated inositol requiring enzyme 1 (p‐IRE1α), phosphorylated eukaryotic initiation factor 2 (p‐eIF2α), CCAAT‐enhancer‐binding protein homologous protein (CHOP) and glucose regulated protein 78 (GRP78), were increased in both U87‐ and U251‐siPLP2 cells compared with levels in U87‐ and U251‐siNC cells (Figure 3B).

Next, we used flow cytometry to determine whether PLP2 knockdown induced apoptosis. SiPLP2 knockdown in both cell lines induced apoptosis in both early (annexin V+/PI−) and late (annexin V+/PI+) stage apoptosis (Figure 3C,D; Figures S3B,C). We confirmed these results using Western blot analyses of apoptosis‐related markers. The levels of cleaved‐caspase 9, cleaved‐caspase 3 and cleaved‐PARP were up‐regulated in U87‐ and U251‐siPLP2‐1 after 48 hours (Figure 3E).

A link between ER stress and apoptosis has been demonstrated previously,^24^, ^25^ and a role for CHOP in ER stress‐induced apoptosis has been established.^26^ To determine whether apoptosis resulted from ER stress, we used two siRNAs to suppress the expression of CHOP in U87 cells infected with a lentiviral shRNA construct targeting PLP2. Expression of PLP2 in U87‐shPLP2 (and U251‐shPLP2) was significantly down‐regulated compared to expression in U87‐shNC (and U251‐shNC), as assessed by Western blot (Figure S1). Cleaved‐caspase 3 and cleaved‐PARP expression in U87‐shPLP2 cells transfected with siCHOP did not increase to the levels observed in U87‐shPLP2 transfected with siNC (Figure 3F and Figure S3D). Collectively, these data show that down‐regulation of PLP2 expression induces ER stress and promotes apoptosis which is partially dependent on CHOP in glioma cells.

### 2.4 Down‐regulation of PLP2 also induces autophagy in glioma cells

**Figure 4:**
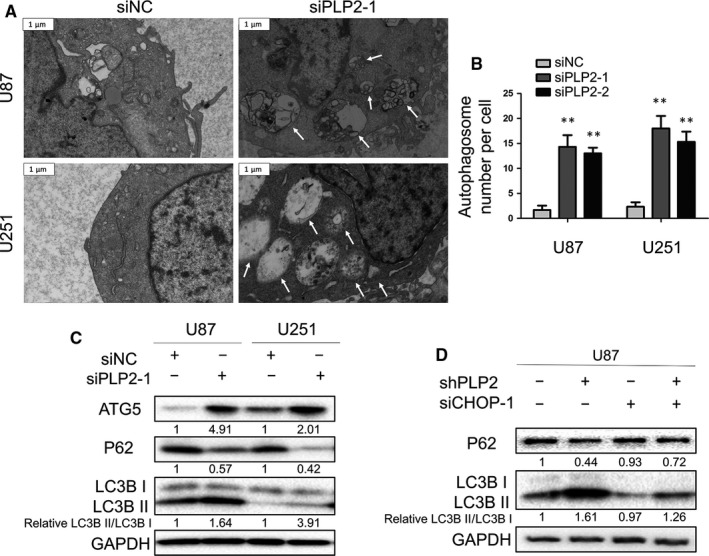
Down‐regulation of PLP2 expression induces autophagy in glioma cells. A, Transmission electron microscopy images showing formation of autophagosomes in U87‐ and U251‐siPLP2‐1 (right) or ‐siNC (left) cells at 48 h after transfection. Representative autophagosomes are highlighted with white arrows. Scale bars = 1 μm. B, Statistical analysis based on the number of autophagosomes per cell. Data are shown as the mean ± SD. **P < .01. C, Western blot analysis to detect autophagy‐related markers ATG5, P62 and LC3B in U87‐ and U251‐siNC or ‐siPLP2‐1 cells. D, Western blot analysis to detect LC3B and p62 levels in U87‐shNC and U87‐shPLP2 cells after transfection with siNC or siCHOP‐1 at 48 h

Previous studies have suggested that ER stress may induce autophagy in various cancers.^24^, ^27^, ^28^ We therefore determined whether PLP2 knockdown was able to induce autophagy in the U87 and U251 cells. Transmission electron microscopy is commonly used to identify the formation of autophagosomes, which is characterized by their double‐membrane structures. Autophagosomes were quantified using TEM and demonstrated that production of autophagosomes was significantly increased in both U87‐ and U251‐siPLP2 cells compared with siNC‐transfected cells (*P* < .001; Figure 4A,B; Figure S3A).

Levels of classical markers associated with autophagy were also assessed in U87‐ and U251‐siPLP2 cells by Western blot. PLP2 knockdown led to increased ATG5 and an increased LC3B‐II/LC3B‐I ratio, but decreased P62. These results were consistent with enhanced autophagic flux (Figure 4C). To further confirm the relationship between ER stress and autophagy induced by PLP2 knockdown, we examined expression of these protein markers for autophagy by Western blot in U87‐shNC and U87‐shPLP2 cells transfected with siNC or siCHOP. The absence of CHOP interfered with the increase in the LC3BII/LC3BI ratio and the reduced levels of p62 induced by shPLP2 (Figure 4D and Figure S4). In conclusion, down‐regulation of PLP2 also induces autophagy which is mediated in part by ER stress‐induced CHOP in glioma cells.

### 2.5 Autophagy inhibition augments apoptotic cell death induced by PLP2 knockdown

**Figure 5:**
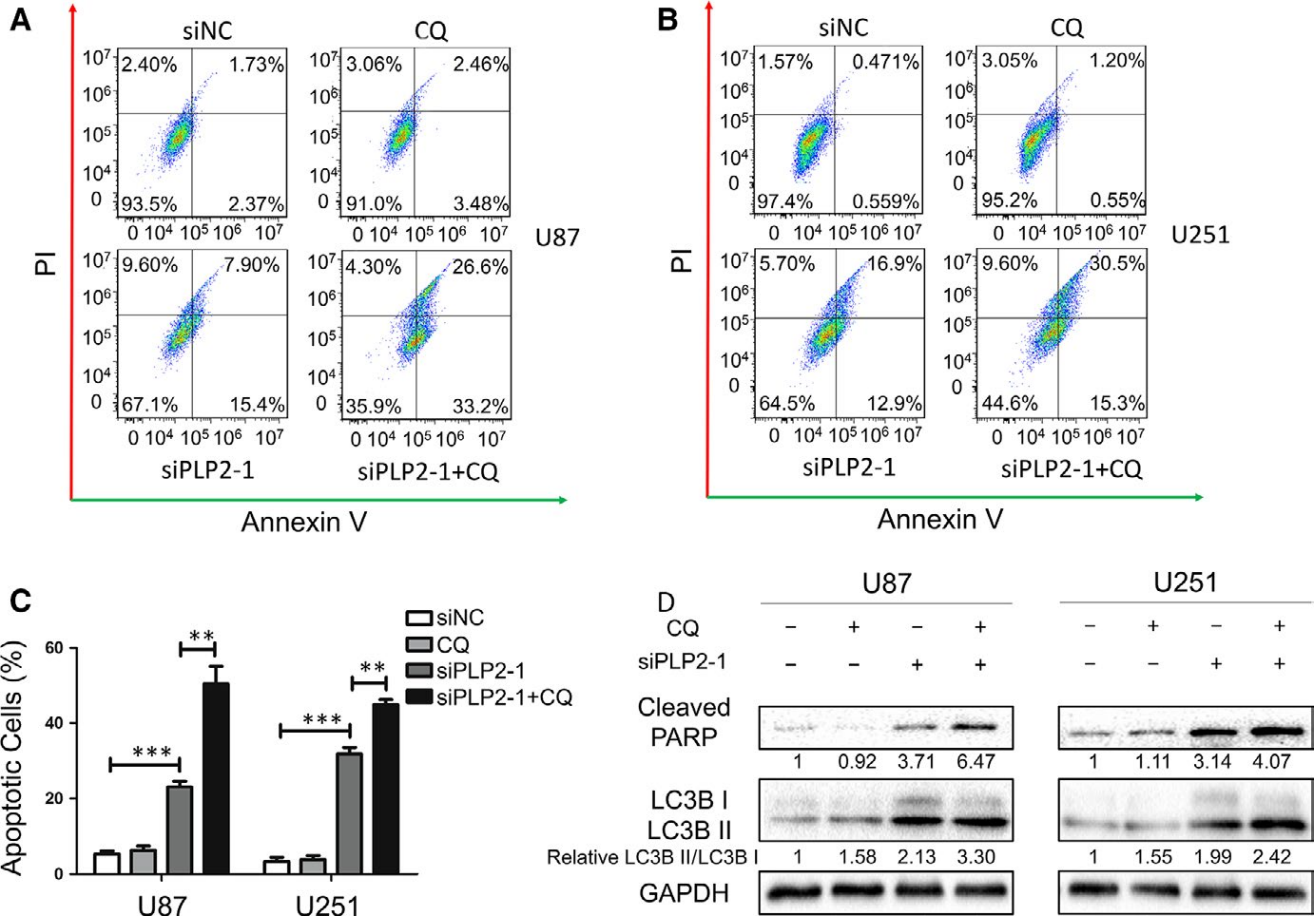
Autophagy inhibition augments apoptotic cell death induced by PLP2 knockdown. A and B, Flow cytometric analysis using annexin V and PI staining to determine apoptosis in U87 and U251 cells pre‐treated with the autophagy inhibitor CQ for 1 h or vehicle control, followed by transfection with siNC or siPLP2‐1. C, Quantification of the percentage of apoptotic cells analysed by flow cytometry in treated U87 and U251 cells. Data are shown as the mean ± SD. **P < .01 and ***P < .001. D, Western blot analysis to detect cleaved‐PARP and LC3B levels in U87 and U251 cells pre‐treated with the autophagy inhibitor CQ or vehicle control for 1 h followed by transfection with siNC or siPLP2‐1

The crosstalk between apoptosis and autophagy has been well demonstrated in other studies.^29^, ^30^ In order to determine whether PLP2 knockdown‐induced autophagy was cytoprotective or cytotoxic, autophagy was inhibited pharmacologically using chloroquine (CQ). Cells were pre‐treated with 10 µmol/L CQ or vehicle control for 1 hours prior to transfection with siPLP2‐1 for 48 hours. Treatment with CQ increased apoptosis in U87‐ and U251‐siPLP2 cells as detected by flow cytometry (Figure 5A,B). Statistical analysis revealed that the percentages of apoptotic cells were significantly increased in the siPLP2‐1 + CQ group compared with the siPLP2‐1+ vehicle control group (Figure 5C). Consistent with these data, the protein levels of cleaved‐PARP were also increased in siPLP2‐1/CQ‐treated cells compared with siPLP2‐1‐treated cells (Figure 5D). Taken together, our results demonstrate that inhibition of autophagy augments apoptotic cell death induced by PLP2 knockdown in U87 and U251 glioma cell lines.

### 2.6 Down‐regulation of PLP2 inhibits glioma growth in vivo

**Figure 6:**
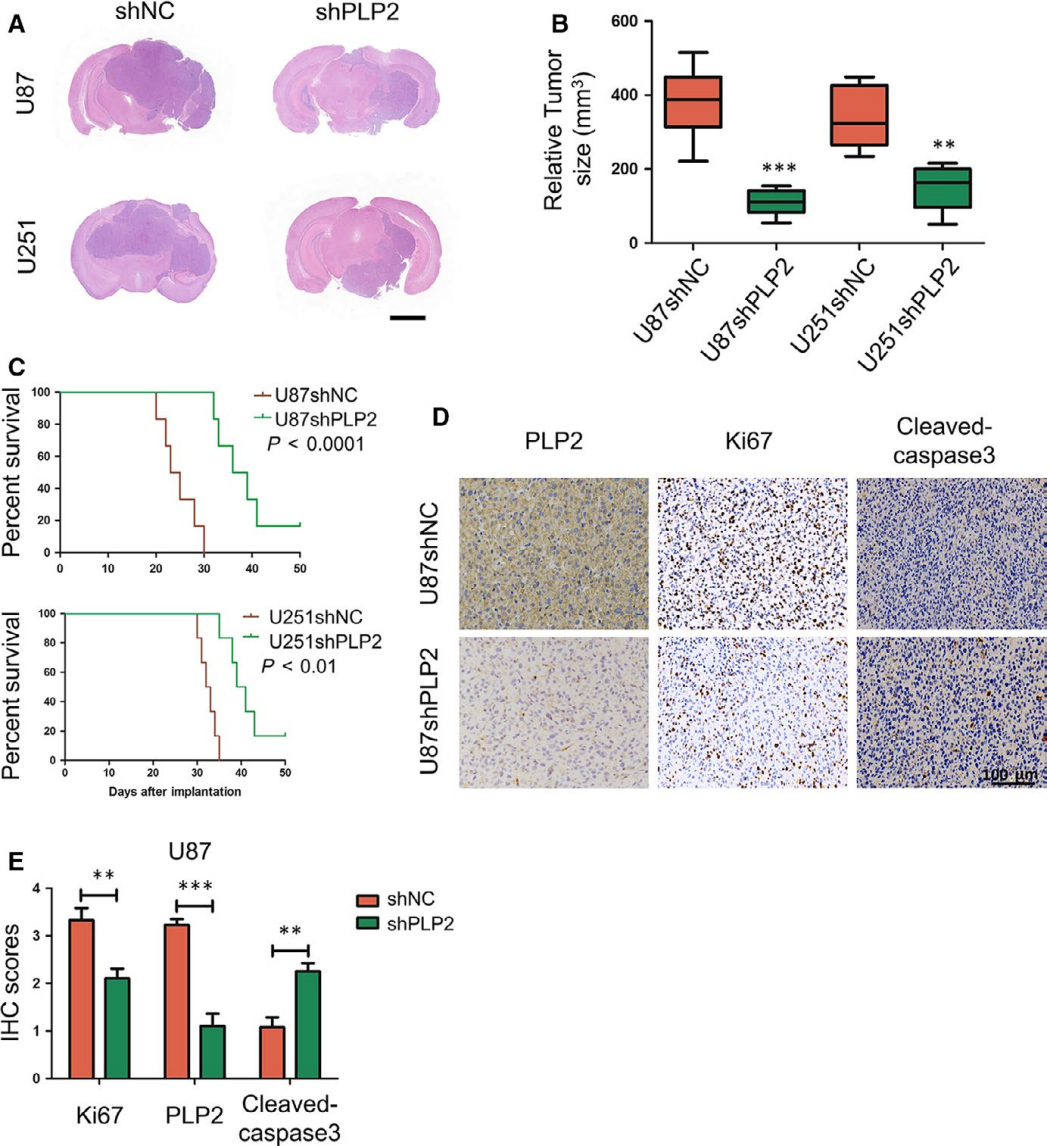
Down‐regulation of PLP2 inhibits glioma cell growth in vivo. A, Representative images of haematoxylin and eosin‐stained sections from the brains of nude mice implanted intracranially with U87‐shNC, U87‐shPLP2, U251‐shNC or U251‐shPLP2 cells. Scale bar = 2 mm. B, Mean relative tumour sizes in the brains of nude mice implanted intracranially with U87‐shNC, U87‐shPLP2, U251‐shNC or U251‐shPLP2 cells. C, Kaplan‐Meier survival analysis of overall survival, and log‐rank analysis to assess the statistical significance of the differences. D, Representative images of immunohistochemical staining for PLP2, Ki‐67 and cleaved‐caspase3 expression in xenograft sections from mice injected with U87‐shNC or U87‐shPLP2 cells. Scale bar = 100 μm. E, Graphic representation of IHC scoring of PLP2, Ki‐67 and cleaved‐caspase3 expression in xenograft sections. All data are presented as the mean ± SD, **P < .01, ***P < .001

To assess the function of PLP2 in vivo, we established orthotopic tumour models by implanting U87‐shNC, U87‐shPLP2, U251‐shNC and U251‐shPLP2 cells intracranially into nude mice. Tumour sizes were decreased in both U87‐ and U251‐shPLP2 cells compared to controls (Figure 6A,B). Overall survival of animals was also significantly prolonged in shPLP2 compared to shNC control groups (*P* < .01, *P* < .001, Figure 6C).

Immunohistochemical staining was performed on sections from U87‐shNC and U87‐shPLP2 xenografts to determine levels of PLP2, Ki‐67 (a marker for proliferation) and cleaved‐caspase 3 (a marker apoptosis). Levels of PLP2 and Ki‐67 were decreased whereas levels of cleaved‐caspase3 were increased in U87‐shPLP2 xenografts compared with U87‐shNC xenografts (Figure 6D,E).

## 3 DISCUSSION

PLP2 has previously been identified as a protein enriched in the colonic epithelium.^31^ PLP2 contains four putative membrane‐spanning α‐helices which multimerize to form ion channels.^3^ In cancer, PLP2 has been reported to have an oncogenic role in melanoma, breast cancer and osteogenic sarcoma.^4^, ^5^, ^6^ In the present study, we found that *PLP2* was highly expressed in high‐grade gliomas relative to low‐grade gliomas and normal brain tissues based on data from three publicly available databases and in our own cohort of clinical samples. Moreover, high *PLP2* expression was associated with poor patient survival.

We further show that down‐regulation of PLP2 inhibits the proliferation of glioma cells in vitro. Moreover, its inhibition also causes reduced intracranial tumour growth in vivo which leads to enhanced overall survival in animals. Our work therefore identifies PLP2 as a potential oncogene in glioma progression and provides a putative rationale for using the protein as a prognostic marker, or as a target in treatment of the disease. A prior study has also suggested an oncogenic role for PLP2 in glioma.^21^ However, our study is the first to establish that PLP2 down‐regulation triggers ER stress‐induced apoptosis and autophagy.

When ER stress is excessive and prolonged, morphological changes are apparent in the ER, and cells will eventually undergo apoptosis.^32^, ^33^ A previous study has shown that PLP2 regulates cellular responses to stimuli that converge towards the ER stress pathway, thus providing strong evidence that reduced PLP2 expression increases susceptibility to ER stress.^7^ Also, it is well established that CHOP and GRP78, which can be regarded as ER stress markers, play an important role in ER stress.^34^ In the present work, TEM demonstrated that PLP2 knockdown results in less distinct ER morphology, a significant up‐regulation of apoptosis‐related markers, and increased expression of ER stress‐related proteins. In addition, CHOP knockdown partially reversed apoptosis induced by PLP2 silencing, as shown by activation of the PERK‐eIF2α‐CHOP pathway.

Autophagy may also be induced by ER stress in gliomas.^35^, ^36^ In our study, the LC3BII/LC3BI ratio and levels of ATG5 were increased while p62 was decreased in our glioma cell lines transfected with siPLP2. These results are consistent with an increase in autophagy flux. Furthermore, autophagy flux induced by PLP2 knockdown was partially reversed via CHOP silencing, indicating that autophagy is also linked to ER stress induction. Our results further show that the use of CQ to inhibit autophagy pharmacologically increased apoptosis in the PLP2‐silenced cells. These results suggested a possible augmentation of PLP2 knockdown‐induced apoptosis through inhibition of autophagy.

In summary, we have shown that down‐regulation of PLP2 increased ER stress‐induced apoptosis, and cotreatment with an autophagy inhibitor further reduced tumour cell survival in vitro. Expression levels of PLP2 may thus be used as a potential future clinical biomarker in GBM, and combined use of PLP2 down‐regulation with autophagy inhibition may have a therapeutic advantage in GBM treatment. However, more studies on the molecular mechanisms underlying PLP2 activity in glioma are warranted before translation of the strategy into clinical practice.

## 4 MATERIALS AND METHODS

### 4.1 Ethics statement

All experiments performed with human samples were approved by the Research Ethics Committee of Shandong University. Informed written consent was obtained from all participants. All animal studies and procedures were approved by the Institutional Animal Care and Use Committee (IACUC) of Shandong University.

### 4.2 Cell lines and cultures

Human glioma cell lines, U87, A172, U251 and T98, were purchased from the Chinese Academy of Sciences Cell Bank. Normal human astrocytes (NHA) were kindly provided by Prof. Rolf Bjerkvig, the Department of Biomedicine, the University of Bergen, Norway. Cells were cultured in Dulbecco's modified Eagle's medium (DMEM, SH30022.01B, Thermo Fisher Scientific) supplemented with 10% foetal bovine serum (10082147 HyClone, GE Healthcare Life Sciences) at 37°C in a humidified incubator containing 5% CO_2_.

### 4.3 Immunohistochemistry

Sections (4 μm) were cut from paraffin‐embedded human and mouse tissues and mounted onto microscope slides. The slides were put into a glass container containing 10 mmol/L citric acid buffer (pH 7.2), and heat‐induced epitope retrieval was performed in a microwave. The sections were blocked with goat serum, incubated with primary antibodies at 4°C overnight (PLP2, 1:200, Ki67, 1:300, Cleaved‐caspase3, 1:300, Cell Signaling Technology), rinsed with PBS and incubated with poly‐HRP secondary antibodies for 30 minutes at room temperature (RT). Visualization was achieved using diaminobenzidine as the substrate, and slides were counterstained with Mayer's haematoxylin. Normal mouse serum was used as a negative control. Staining of cancer cells was scored as follows: 0, no staining; 1, weak staining in <50% cells; 2, weak staining in ≥50% cells; 3, strong staining in <50% cells; and 4, strong staining in ≥50% cells.

### 4.4 Western blot analysis

Cells were harvested, rinsed with cold PBS, and lysed using RIPA buffer containing a protein inhibitor cocktail (Thermo Fisher Scientific). Protein lysates (20 µg) were electrophoresed on 10% SDS‐PAGE and transferred onto polyvinylidene difluoride (PVDF) membranes (ISEQ00010 0.22 μm, Millipore). Membranes were blocked in 5% skim milk blocking buffer for 1 hour at RT with 5% skim milk in TBST (20 mmol/L Tris‐HCL (pH 8.0), 137 mmol/L NaCl and 0.1% Tween‐20 (or with 5% BSA in TBST for phospho‐proteins) and incubated with primary antibodies overnight at 4°C. Membranes were incubated with the following antibodies: PLP2, p‐IRE1α (Ser724, Abcam), ACTB, p‐PERK (Thr 980), PERK, IRE1α, p‐eIF2α (Ser51), eIF2α, CHOP, GRP78, cleaved‐caspase9, caspase9, cleaved‐caspase3, caspase3, cleaved‐PARP, PARP, ATG5, P62, LC3B, GAPDH (Cell Signaling Technology). For detection, membranes were washed with TBST and incubated with the appropriate horseradish peroxidase (HRP)‐conjugated secondary antibodies, antimouse immunoglobulin G (IgG) or anti‐rabbit IgG (1:5000, Santa Cruz Biotechnology) for 1 hour at RT. The protein bands were visualized using Millipore's enhanced chemiluminescence (ECL) and detection system (ChemiDoc Touch, Bio‐Rad). Bands were quantified and normalized relative to the loading control. Fold differences are labelled under each image.

### 4.5 Small interfering RNA transfections

U87 and U251 cells were transfected with gene‐specific siRNAs or negative control siRNA, synthesized by GenePharma, for 48 hours using Lipofectamine 2000 (Thermo Fisher Scientific, 11668‐027) according to the manufacturer's protocol. Cells were seeded at a density of 3 × 10^5^ cells/well in 6‐well plates. The siRNA duplexes were transfected into cells when the cells were 70‐80% confluent. The following siRNA sequences were used to target the RNAs indicated: siNC: 5′‐UUCUCCGAACGUGUCACGUTT‐3′

siPLP2‐1:5′‐CCCUGUCGGUGAUUGAGAUTT‐3′

siPLP2‐2:5′‐CCAAGAUACCAUUCAUCAATT‐3′

siCHOP‐1:5′‐AAGAACCAGCAGAGGUCACAA‐3′

siCHOP‐2:5′‐ACCAAGGGAGAACCAGGAAAC‐3′

Western blot analysis was used to evaluate siRNA knockdown efficiency.

### 4.6 Cell viability and proliferation assays

The Cell Counting Kit‐8 (CCK‐8, CK04‐500, Dojindo) was used to assess cell viability. Cells (1.0 × 10^4^ cells/well) were seeded into 96‐well plates and incubated at 37°C overnight. After treatment, 10 μL of CCK‐8 in 100 μL of serum‐free DMEM was added to replace the original medium. Cells were then incubated for an additional 4 hours at 37°C. The optical absorbance at 450 nm was detected using a microplate reader (Bio‐Rad). Proliferation was assessed using the EdU incorporation assay according to the manufacturer's protocol (C103103, Ribobio). Briefly, cells were seeded onto 24‐well plates at a density of 5.0 × 10^4^ cells per well. After treatment, EdU was incorporated into proliferating cells, which is detected through a catalysed reaction with a fluorescently labelled azide. Labelled cells were examined under fluorescence microscopy, and the number of EdU‐positive cells was counted in three random fields.

### 4.7 Transmission electron microscopy (TEM)

Cells were fixed with 4% glutaraldehyde and post‐fixed with 1% OsO_4_ in 0.1 mol/L cacodylate buffer containing 0.1% CaCl_2_ at 4°C for 2 hours. Samples were stained with 1% Millipore‐filtered uranyl acetate, dehydrated in increasing concentrations of ethanol, and infiltrated and embedded in epoxy resin. Ultrathin sections were cut and stained with uranyl acetate and lead citrate. Electron photomicrographs were obtained, using a transmission electron microscope (JEM‐1200EX II, JEOL).

### 4.8 Apoptosis assay

Cells seeded in 6‐well plates were detached with 0.25% trypsin, washed, resuspended in binding buffer and incubated with annexin V‐FITC antibody (BD Biosciences) according to the manufacturer's instructions. Apoptotic cells were detected by flow cytometry (ACEA Biosciences), and the corresponding results were analysed using the software Flowjo, version 7.6.5, (Tree Star).

### 4.9 Lentiviral transduction

Lentiviral vectors expressing human shRNA targeting PLP2 (shPLP2, LV2017‐18615, GenePharma Shanghai) or scrambled‐control (shNC) were used to generate stable cell clones expressing shPLP2 or a nonspecific shRNA as the control. Transfected clones were selected using 1 mg/mL of puromycin (Selleckchem). Western blot analysis was used to evaluate shRNA knockdown efficiency.

### 4.10 Intracranial xenograft model

Twenty‐four athymic, male mice (4 weeks old, 20‐30 g; Shanghai SLAC Laboratory Animal Co., Ltd) were randomly divided into the following four groups: U87‐shNC (n = 6), U87‐shPLP2 (n = 6), U251‐shNC (n = 6), and U251‐shPLP2 (n = 6). The mice were anesthetized with chloral hydrate and secured on a stereotactic frame. A longitudinal incision was made in the scalp and a 1 mm‐diameter hole was drilled 2.5 mm lateral to the bregma. Cells (2 × 10^5^) in 20 μL of serum‐free DMEM were implanted 2.5 mm into the right striatum using a Hamilton syringe. Animals which displayed symptoms such as severe hunchback posture, apathy, decreased motion or activity, dragging legs, or drastic loss of body weight were euthanized by cervical dislocation. Excised tumour tissues were formalin‐fixed, paraffin‐embedded, and sectioned. Sections were either stained with haematoxylin and eosin (HE) or used for IHC. The relative tumour size was calculated as 0.5 × A × B^2^, where A represents the length of the tumour, and B represents the width of the tumour obtained from HE sections.

### 4.11 Statistical analysis

All statistical analyses and experimental graphs were performed using GraphPad Prism 5 software. The results are presented as the mean ± the standard deviation (SD). Mean values were compared using Student's *t* test for paired data. Survival curves were estimated with the Kaplan‐Meier method and compared using the log‐rank test. All in vitro experiments were repeated independently at least three times. All tests were two‐sided, and *P*‐values determined from different comparisons <.05 were considered statistically significant and are indicated as follows: ns‐not significant, *P < .05, **P < .01, ***P < .001.

## CONFLICT OF INTEREST

The authors declare no potential conflicts of interest.

## AUTHOR CONTRIBUTIONS

XL and JW designed the study. ZF, WZ and JWW performed the experiments; QQ, MH, YK, YH, YZ and ABC contributed to analysis and interpretation of data. BH, AJC, DZ, WL, QZ, FT and RB contributed to the writing of the manuscript. FT contributed to the correction and submission of the manuscript. ZF prepared all figures and tables. All authors reviewed the manuscript and approved the final versions.

## Data Availability

The data that support the findings of this study are available from the corresponding authors upon reasonable request.
