# Supplementary material for: Reduced expression of proteolipid protein 2 increases ER stress‐induced apoptosis and autophagy in glioblastoma
Source: J Cell Mol Med. 2019 Nov 28;24(5):2847–56. doi: 10.1111/jcmm.14840 (PMC7077595; doi:10.1111/jcmm.14840)
Supplement: Supplementary file 1 [file JCMM-24-2847-s001.docx]

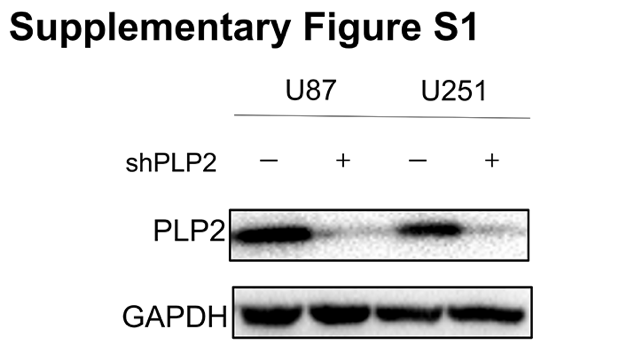


**Supplementary Figure S1**

Western blot analysis to detect knockdown efficiency of lentiviral constructs expressing shPLP2 in infected U87 and U251 cells.


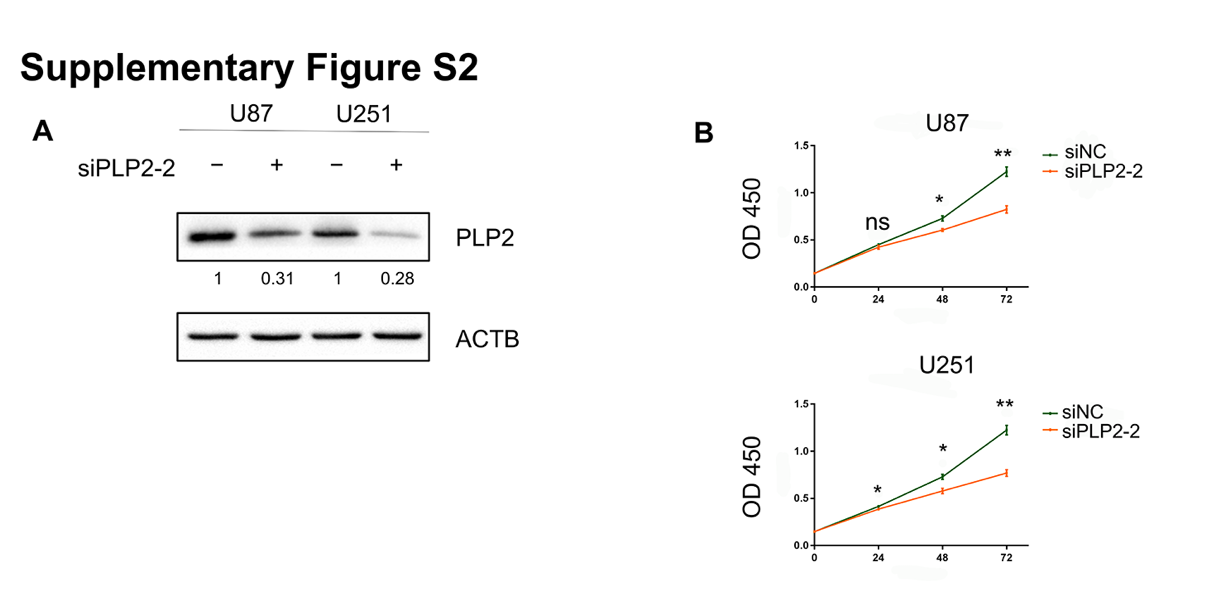


**Supplementary Figure S2**

**(A**) Confirmation of knockdown efficiency of siPLP2-2 in U87 and U251 cells, as determined by western blot analysis.

(**B**) Cell viability of U87- and U251-siNC and -siPLP2-2 cells determined with CCK-8 assays. ns-not significant, * *P* < 0.05, and ** *P* < 0.01.


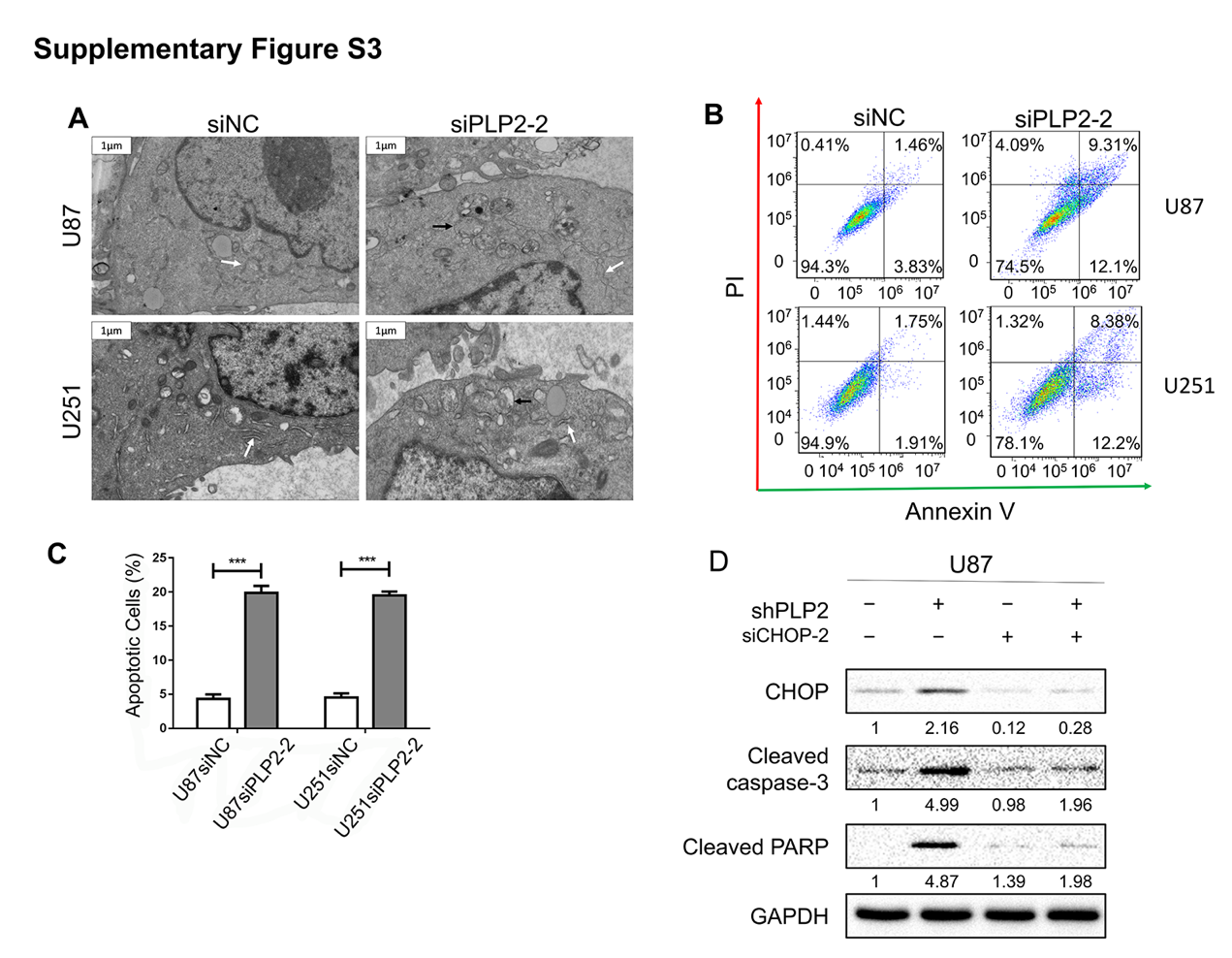


**Supplementary Figure S3**

**(A**) Transmission electron microscopy images showing dilated ER and formation of autophagosomes in U87- and U251-siPLP2-2 cells (right), compared with -siNC cells 48 h after transfection. Representative ER are highlighted with white arrows and autophagosomes with black arrows. Scale bars = 1 μm.

(**B**) Apoptosis in U87- and U251-siNC or -siPLP2-2 48 h after transfection, as assessed by flow cytometry.

(**C**) Quantification of the percentage of apoptotic cells analyzed by flow cytometry. * *** *P* < 0.001.

(**D**) Western blots showing the levels of cleaved-caspase3 and cleaved-PARP levels in U87-shNC and U87-shPLP2 cells transfected with siNC or siCHOP-2.


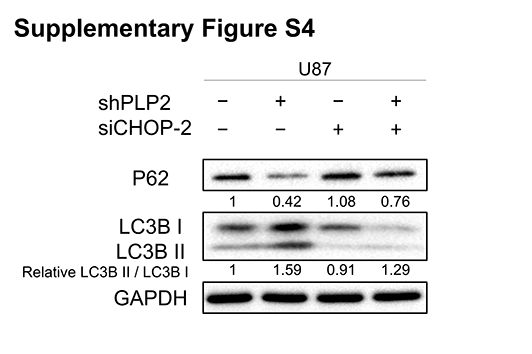


**Supplementary Figure S4**

Western blot analysis to detect LC3B and p62 levels in U87-shNC and U87-shPLP2 cells 48 h after transfection with siNC or siCHOP-2.


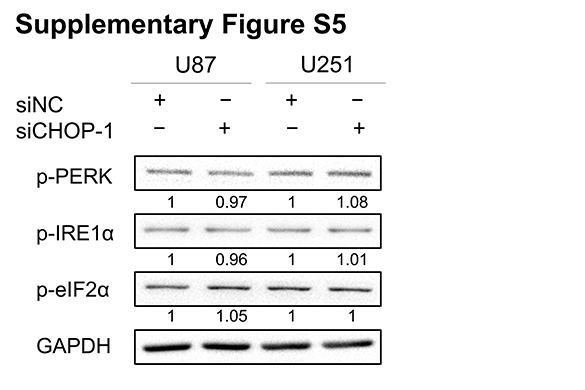


**Supplementary Figure S5**

Western blots showing the levels of the ER stress related markers p-PERK, p-IRE1α, and p-eIF2α in U87 and U251 cells 48 h after transfection with siNC or siCHOP-1.
